# Cognitive, Behavioral, and Learning Profiles of Children with Above-Average Cognitive Functioning: Insights from an Italian Clinical Sample

**DOI:** 10.3390/children12070926

**Published:** 2025-07-13

**Authors:** Daniela Pia Rosaria Chieffo, Valentina Arcangeli, Valentina Delle Donne, Giulia Settimi, Valentina Massaroni, Angelica Marfoli, Monia Pellizzari, Ida Turrini, Elisa Marconi, Laura Monti, Federica Moriconi, Delfina Janiri, Gabriele Sani, Eugenio Maria Mercuri

**Affiliations:** 1 Clinical Psychology Unit, Fondazione Policlinico Universitario Agostino Gemelli IRCCS, Largo Francesco Vito 1, 00168 Rome, Italy; danielapiarosaria.chieffo@policlinicogemelli.it (D.P.R.C.); valentina.arcangeli@policlinicogemelli.it (V.A.); settimigiulia@gmail.com (G.S.); monia.pellizzari2@studio.unibo.it (M.P.); elisa.marconi@policlinicogemelli.it (E.M.); laura.monti@policlinicogemelli.it (L.M.); federica.moriconi@policlinicogemelli.it (F.M.); 2 Complex Operational Unit of Child Neuropsychiatry, Fondazione Policlinico Universitario Agostino Gemelli IRCCS, 00168 Rome, Italy; ida.turrini@policlinicogemelli.it (I.T.); eugeniomaria.mercuri@policlinicogemelli.it (E.M.M.); 3 Department Women Children and Public Health, Università Cattolica del Sacro Cuore, 20123 Rome, Italy; 4 Faculty of Medicine and Surgery, Department of Health Science and Public Health, Catholic University of the Sacred Heart, 20123 Rome, Italy; valentina.massaroni@unicatt.it; 5 Complex Operational Unit of Clinical and Emergency Psychiatry, Fondazione Policlinico Universitario Agostino Gemelli IRCCS, 00168 Rome, Italy; angelica.marfoli@guest.policlinicogemelli.it (A.M.); delfina.janiri@policlinicogemelli.it (D.J.); gabriele.sani@policlinicogemelli.it (G.S.); 6 Department of Neuroscience, Section of Psychiatry, Università Cattolica del Sacro Cuore, 20123 Rome, Italy

**Keywords:** above-average cognitive functioning, cognitive assessment, twice-exceptionality, executive functions, internalizing symptoms, learning difficulties, Wechsler scales, MT battery, child psychology, clinical neuropsychology

## Abstract

Background/Objectives: Children with above-average cognitive functioning often present complex developmental profiles, combining high cognitive potential with heterogeneous socio-emotional and learning trajectories. Although the cognitive and behavioral characteristics of giftedness have been widely studied in Anglophone countries, evidence remains limited in Southern Europe. This study aimed to investigate the cognitive, academic, and emotional–behavioral profiles of Italian children and adolescents with above-average cognitive functioning, using an inclusive, dimensional approach (IQ > 114). Methods: We analyzed a cross-sectional sample of 331 children and adolescents (ages 2.11–16.5 years), referred for clinical cognitive or behavioral evaluations. Participants were assessed using the WPPSI-III or WISC-IV for cognitive functioning, the MT battery for academic achievement, and the Child Behavior Checklist (CBCL) for emotional and behavioral symptoms. Comparative and correlational analyses were performed across age, gender, and functional domains. A correction for multiple testing was applied using the Benjamini–Hochberg procedure. Results: Gifted participants showed strong verbal comprehension (mean VCI: preschoolers = 118; school-aged = 121) and relative weaknesses in working memory (WM = 106) and processing speed (PS = 109). Males outperformed females in perceptual reasoning (PR = 121 vs. 118; *p* = 0.032), while females scored higher in processing speed (112 vs. 106; *p* = 0.021). Difficulties in writing and arithmetic were observed in 47.3% and 41.8% of school-aged participants, respectively. Subclinical internalizing problems were common in preschool and school-aged groups (mean CBCL T = 56.2–56.7). Working memory negatively correlated with total behavioral problems (r = −0.13, *p* = 0.046). Conclusions: These findings confirm the heterogeneity of gifted profiles and underscore the need for personalized educational and psychological interventions to support both strengths and vulnerabilities in gifted children. Caution is warranted when interpreting these associations, given their modest effect sizes and the exploratory nature of the study.

## 1. Introduction

The concept of intellectual giftedness (IG) has been a cornerstone of psychological and educational research, often associated with exceptional intellectual capabilities and achievements across diverse domains, such as academics, the arts, and leadership [1,2]. Traditionally, intellectual giftedness has been defined by an intelligence quotient (IQ) exceeding two standard deviations above the mean (≥130) [3,4]. The most widely used standardized measures to identify IG are the Wechsler intelligence scales [3,5,6], which allow for the estimation of individual IQ and for which intelligence is a set of cognitive abilities, related to planning, learning speed, reasoning, problem-solving, and understanding complex ideas [4]. However, evolving frameworks underscore the multifaceted nature of giftedness, which extends beyond standardized IQ measures to include emotional, social, and behavioral dimensions [7,8].

Recent studies have emphasized the role of socio-cultural and environmental factors in shaping the expression and challenges of giftedness, particularly in relation to emotional adjustment and developmental outcomes [9].

A unique aspect of giftedness is asynchronous development, where advanced cognitive abilities coexist with uneven emotional and social maturity, potentially leading to socio-emotional challenges. These challenges include anxiety, social withdrawal, low self-esteem, and perfectionism [10,11]. Moreover, the phenomenon of twice-exceptionality, where giftedness coexists with conditions such as learning disabilities or ADHD, poses unique challenges for both identification and support. This interplay can obscure underlying difficulties, resulting in underdiagnosis and delayed interventions [12,13]. Studies have shown that gifted children with ADHD exhibit distinct profiles, often presenting stronger coping strategies that mask their symptoms, underlining the need for refined diagnostic tools [14,15]. Understanding this complexity is crucial for tailoring effective interventions. From a theoretical standpoint, this study adopts a developmental and non-pathologizing perspective of giftedness. Emotional and behavioral vulnerabilities—such as anxiety, perfectionism, or social withdrawal—are not necessarily seen as clinical deviations, but rather as potential outcomes of asynchronous development and heightened sensitivity, especially in unsupportive environments [10,11]. This framework encourages a nuanced understanding of the interplay between cognitive advancement and emotional regulation.

Despite substantial research on the cognitive and emotional aspects of giftedness, gaps remain in understanding how these dimensions interact within specific populations. Existing studies often highlight cognitive strengths, such as verbal reasoning, or explore emotional vulnerabilities, but rarely consider these elements in tandem with learning outcomes [16,17]. Furthermore, most investigations are conducted in Anglophone contexts, with limited exploration of Mediterranean populations, such as Italian children and adolescents. In particular, studies involving Southern European or Mediterranean populations remain scarce, despite growing evidence that socio-cultural and educational factors significantly shape the developmental trajectories of gifted children [9,18,19,20,21,22]. To our knowledge, no previous research has systematically explored cognitive, academic, and emotional profiles within an Italian clinical cohort of intellectually gifted children and adolescents. This is a relevant gap, considering the specificities of the Italian educational system, the role of cultural values, and the known challenges in recognizing twice-exceptionality in real-world clinical practice.

Moreover, our study adopts a dimensional and inclusive operationalization of giftedness (IQ > 114), as recommended by recent international guidelines [7,8,23], allowing for the identification of high-functioning individuals who may not meet the strict IQ ≥ 130 cut-off but nonetheless present with exceptional cognitive potential alongside academic or socio-emotional vulnerabilities. This approach reflects the complexity of clinical referrals and aligns with current efforts to capture a broader spectrum of giftedness beyond traditional, elitist definitions.

These gaps hinder a comprehensive understanding of how socio-cultural contexts influence the manifestation and challenges of giftedness. In addressing these gaps, our study focuses on a clinically referred cohort of Italian children and adolescents with above-average cognitive functioning (IQ > 114), a group often overlooked by research focusing exclusively on strict giftedness criteria (IQ ≥ 130). This operationalization allows for the investigation of cognitive, academic, and emotional profiles in children who present both high intellectual potential and real-world developmental challenges, including learning or socio-emotional difficulties. Furthermore, our study extends current knowledge by providing data from a Southern European context, where cultural, educational, and clinical dynamics may differ significantly from those observed in Anglophone populations.

Emerging evidence suggests that cultural and regional differences significantly influence the experiences of twice-exceptional children, further underscoring the importance of localized research efforts [13,18].

Moreover, recent empirical studies have highlighted how executive functioning profiles [19], environmental factors such as school support systems [20], and even digital learning contexts [21] may affect the trajectories of intellectually gifted children. A systematic integration of these findings within non-Anglophone populations remains limited, especially in Southern Europe, where sociocultural expectations and educational resources differ significantly from Anglo-Saxon models [22].

The primary objectives of this research are as follows:

Describe the cognitive and learning profiles of a cohort of Italian children and adolescents with above-average cognitive functioning, referred for clinical evaluation.

Investigate the associations between cognitive abilities (e.g., working memory, processing speed) and learning outcomes.

Examine the relationship between emotional–behavioral problems and high cognitive potential, exploring internalizing and externalizing tendencies across developmental stages.

By defining these objectives, the study aims to provide a holistic understanding of the profiles of children with above-average cognitive functioning, linking cognitive abilities, learning achievements, and emotional characteristics within a Mediterranean population.

This study seeks to address these limitations by analyzing the cognitive, emotional, and learning profiles of Italian children and adolescents with above-average cognitive functioning. By adopting an inclusive operationalization of above-average cognitive functioning (IQ > 114), this research acknowledges a broader spectrum of intellectual potential, including high-functioning individuals who may not meet stricter definitions [8,23]. This threshold, although lower than the conventional cut-off of IQ ≥ 130, reflects a dimensional and inclusive approach to the identification of intellectual potential, particularly suited for clinical and educational settings. Previous authors have emphasized the utility of broader operationalizations (e.g., IQ > 115) to capture individuals who may present with high potential but also experience significant challenges in academic or socio-emotional domains [7,8,23].

This approach allows for a nuanced exploration of the relationships between cognitive abilities, learning outcomes, and emotional–behavioral characteristics. Furthermore, recent contributions highlight how perfectionism and socio-emotional challenges often co-occur in high-functioning populations, with variations observed across cultural and environmental contexts [24]. Notably, longitudinal studies conducted in post-pandemic contexts [25] suggest an increased variability in academic outcomes and well-being among gifted students, further reinforcing the need for updated, multidimensional assessments. This study is grounded in the view that cognitive potential cannot be fully understood through IQ scores alone. It builds on and extends current literature by combining cognitive, academic, and emotional–behavioral data within a Mediterranean context. Unlike previous work that often isolates these domains or adopts a deficit-oriented lens, we aim to highlight the complex, multidimensional nature of high cognitive functioning—its strengths, vulnerabilities, and implications for intervention.

The main research questions addressed in this study are therefore as follows:

- What are the cognitive strengths and weaknesses of cognitively gifted children and adolescents with above-average cognitive functioning, and how do these profiles vary by age and gender?
- How are cognitive profiles related to specific learning outcomes, such as reading, writing, and mathematical skills?
- What emotional and behavioral characteristics are prevalent in this population, and how do they interact with cognitive and learning profiles?
- What practical insights can be derived to inform educational and clinical interventions for gifted children, particularly those with learning or emotional difficulties?

By addressing these questions, this study aims to contribute novel insights into the developmental pathways of children with above-average cognitive functioning. The findings are intended to support the development of targeted educational and clinical interventions, ultimately helping these children reach their full potential across diverse contexts.

## 2. Materials and Methods

### 2.1. Participants

This was a cross-sectional study. The study involved 331 children and adolescents aged 2.11 to 16.5 years (mean age = 8.68, standard deviation SD = 3.4), referred to the Clinical Psychology Unit of “Fondazione Policlinico Universitario Agostino Gemelli IRCCS” in Rome, Italy, between 2017 and 2024. Males constituted 66.1% (*n* = 219/331) of the sample, while females constituted 33.8% (*n* = 112/331). The preschool subgroup comprised 22.3% of participants (*n* = 74/331), while the school-aged subgroup included 77.7% (*n* = 257/331). Table 1 summarizes the demographic and clinical characteristics of the sample. This was a clinical sample, and healthcare professionals or educators referred all participants for cognitive and/or emotional–behavioral evaluations. Inclusion criteria were as follows: an IQ score > 114 as measured by standardized Wechsler scales, indicating above-average cognitive functioning, and referral for psychological or educational assessment related to cognitive development. Exclusion criteria were as follows: presence of known neurological conditions, severe psychiatric disorders (e.g., autism spectrum disorder, major depressive disorder), or insufficient language ability to complete the assessments. Although no formal diagnoses of neurological or psychiatric disorders were present, children were referred due to emerging concerns in developmental, cognitive, or behavioral functioning. This justifies the clinical nature of the sample, which reflects a population at risk or in need of in-depth evaluation rather than a general community cohort.

The institutional ethics committee of the Catholic University of Sacred Heart, Rome, Italy, approved the study (IRB number ID 5234) and all procedures were conducted in accordance with the ethical standards of the institutional and national research committee and with the 1964 Helsinki Declaration and its later amendments. Informed consent was obtained from all parents or legal guardians, and assent was obtained from children capable of providing it.

### 2.2. Procedure

Participants were recruited consecutively through clinical referrals. Group sizes were determined based on clinical availability rather than a priori power analysis. All assessments were conducted individually in a quiet clinical setting, following standardized administration protocols. The evaluation sessions included cognitive testing, academic achievement assessments (for school-aged participants), and parent-completed behavioral questionnaires. For children aged 6–7.3 years, the choice between the WPPSI-III or WISC-IV was based on school placement, in accordance with developmental appropriateness and clinical guidelines. All raw scores were converted to standardized values using the corresponding Italian normative tables. Behavioral observations, including the presence of restless behavior, were systematically recorded during testing sessions. Each evaluation was administered by trained psychologists specializing in developmental assessment, who underwent standardized training to ensure consistency.

### 2.3. Measures

First, we collected information on the demographic variables of age and sex of the sampled children and adolescents. In addition, we collected information about the presence of restlessness behavior during the evaluation. “Restless behavior” was defined as observable motor activity (e.g., fidgeting, difficulty sitting still) during the assessment session, documented by trained assessors using standardized behavioral checklists.

Possible confounding factors such as socio-economic level or the presence of associated disorders were not included in the analysis due to the nature of the clinical sample. However, the sample consisted mainly of children from urban Italian families. Parent demographics, including education level and employment status, were not systematically collected but were considered during clinical evaluations.

All included subjects underwent an assessment of cognitive level and emotional and behavioral problems. For school-aged participants (grade 1 onwards), additional assessments were conducted to evaluate reading, writing, and calculation skills.

#### 2.3.1. Cognitive Level Assessment

To assess the cognitive level of the children we administered the age-appropriate Wechsler scales.

‘Wechsler Preschool and Primary Scale of Intelligence—III (WPPSI-III)’ was administered to children aged 2.6 to 7.3 years, provided they had not yet entered primary education [6,26]. The WPPSI-III provides a verbal intellectual quotient (VIQ), performance intellectual quotient (PIQ), and total intellectual quotient (TIQ). A processing speed quotient (PSQ) for the age group of 4 to 7.3 years can also be derived. In addition, a General Language Index (GLI) can be derived for both age groups.

‘Wechsler Intelligence Scale for Children—Fourth Edition (WISC-IV)’ was administered to children aged 6 to 16.5 years, provided they were enrolled in primary school [3,27]. The WISC-IV provides verbal comprehension (VC), perceptual reasoning (PR), working memory (WM), and processing speed (PS) subscales. The sum of the subtests of the various subscales provides a total intellectual quotient (IQ).

Raw scores were converted to standardized scores for analysis, following manual guidelines [3,6]. The raw scores obtained in each subtest were converted via appropriate tables into weighted scores with a mean of 10 and a standard deviation of 3. The weighted scores obtained were then summed up and converted via appropriate tables into the corresponding quotients with a standardized mean of 100 and a standard deviation of 15.

Reliability of the measures was established using previously published effect sizes, as documented in the WPPSI-III and WISC-IV manuals [3,6].

#### 2.3.2. Assessment Tools and Procedures for School Learning Skills

The group of school-age children and adolescents was subjected to the appropriate tests according to the class they attended, assessing their learning abilities in reading, writing, and calculation.

About reading, the MT-3 Clinical Battery (6–14 years) [28] and the MT-3 Advanced Clinical Battery (14–16 years) [29] were administered to assess reading speed, accuracy, and comprehension.

Which allow for the in-depth assessment, with a single instrument and in a short time: reading and text comprehension skills from primary school up to the second year of secondary school. Text reading and reading comprehension tests were administered. The proposed passages differentiated by class and period of the school year assessed reading comprehension, correctness, and speed.

When necessary, according to age, some tests from the adolescent learning assessment battery for secondary school ‘MT-16-19 reading and writing tests’ [30] were used. Passage reading was administered from which two scores were obtained, one for correctness and one for reading speed, and passage comprehension.

About writing, the Piece Dictation Test of the BVSCO-2 Writing and Spelling Competence Assessment Battery [31] was used. The BVSCO-2 is a comprehensive tool for assessing all aspects involved in learning to write from grade 1 in primary school to grade 3 in secondary school. The score is obtained from the number of spelling errors committed. For children attending school years after grade 3, the dictation test of a text from MT-3 Advanced Clinical or MT-16-19 was used according to the appropriate class.

Finally, the written arithmetic operations test of the ‘AC-MT 6-11’ [32] and ‘AC-MT 11-14’ [33] calculation battery was used to assess mathematical ability. This battery provides standardized evaluation of numerical and calculation skills. For subjects over the age of 14, the MT-3 Advanced Clinical or MT-16-19 arithmetic test was used according to the appropriate class.

The raw scores obtained in each test administered were compared with the reference tables of the class attended and allowed each subject to be placed in one of four performance bands: ‘criterion fully achieved’, ‘sufficient performance’, ‘demand for attention’, and ‘demand for immediate intervention’. The first two bands were within the norm, while the second two were at the lower end of the norm and below the norm.

#### 2.3.3. Emotional and Behavioral Problems Assessments

Child Behavior Checklist (CBCL) is a useful tool in our clinical practice for the assessment of emotional and behavioral problems in children included in the Achenbach System of Empirically Based Assessment (ASEBA) [34].

It is a parent-report assessment tool in two versions according to age groups, in which parents indicate through a 3-point Likert scale from 0 to 2 (0 = not true/absent, 1 = somewhat or sometimes true/occurring, 2 = very true or often true/occurring) the frequency of a series of problems in the last 6 months. The raw scores are then converted into t-scores according to gender and age.

The CBCL for ages 1 ½–5 consists of 99 items that are grouped into the following syndromic subscales: Emotional Reactivity, Anxiety/Depression, Somatic Disturbances, Withdrawn, Attention Problems, Aggressive Behavior and Sleep Problems. The 7 empirical subscales can lead to the 3 main scales: Externalization, Internalization and Total Problems.

CBCL for Ages 6–18 consists of 113 items that are grouped into eight specific syndromic subscales relating to various possible clinical pictures: anxiety/depression, withdrawal/depression, somatic complaints, social problems, thinking problems, attention problems, rule-breaking behavior, aggressive behavior, and other problems. The 8 empirical subscales can lead to the 3 main scales: Externalization, Internalization and Total Problems.

We can interpret the t-scores of all subscales as follows: ≤64 = normal; 65–69 = borderline; ≥70 pathological. Furthermore, we can interpret the t-scores of the main subscales according to the following range: ≤59 = in the normal range; 60–64 = borderline; ≥65 pathological.

### 2.4. Statistical Analysis

We calculated descriptive statistics for quantitative variables [median, interquartile range (IQR), mean, standard deviation (SD)] and qualitative variables (per cent frequencies). We tested the quantitative variables for normal distribution using the Shapiro–Wilk Test.

To carry out the analyses, we considered three groups of subjects separately: those who had been administered the WPPSI-III the 2.6–3.11 years age version, those who had taken the WPPSI-II the 4–7.3 years age version and those who had been administered the WISC-IV.

We used T-tests and ANOVA or the Mann–Whitney U Test and the Kruskal–Wallis test depending on the nature of each variable to examine group differences by age, gender and performance range in which the subjects placed themselves on the school learning tests. About the school learning tests, we made comparisons by dividing the subjects into two groups, the one with scores within the norm comprising the performance ranges ‘criterion fully achieved’ and ‘sufficient performance’ and the one with scores below the norm comprising the performance ranges ‘demand for attention’ and ‘demand for immediate intervention’.

We used Pearson’s and Spearman’s correlations to explore associations between cognitive scores and behavioral/emotional outcomes. As previously indicated, no correlation analyses were conducted between cognitive scores and school learning outcomes.

A two-tailed *p*-value of <0.05 was considered statistically significant. Missing values accounted for less than 5% of the data and were addressed using pairwise deletion, ensuring the inclusion of all available data for each analysis. All analyses were performed using the SPSS version 21.0 software package (SPSS Inc., Chicago, IL, USA).

A correction for multiple testing was applied to control for the false discovery rate (FDR), using the Benjamini–Hochberg procedure. This adjustment was conducted for group comparisons and correlational analyses involving cognitive, academic, and behavioral variables. Adjusted *p*-values (q-values) are reported, with statistical significance considered at q < 0.05.

A post hoc power analysis was conducted to estimate the detectable effect sizes given our sample. The study had sufficient power (≥0.73) to detect moderate group differences (d ≈ 0.5) and high power (≈0.90) to detect moderate correlations (r ≈ 0.3). However, the power to detect small or small-to-moderate effects was limited, suggesting that non-significant findings for weak associations should be interpreted with caution.

## 3. Results

### 3.1. Clinical Characteristics

Restless behavior was noted in 19.6% of participants (*n* = 65/331). Specifically, in the preschool group with ages 2.6–3.11 years, the presence of restlessness behavior was found in 57% of the children (*n* = 12/21), in the group with ages 4–7.3 years in 33.9% of the children (*n* = 18/53) and the school-age group in only 14% of the subjects (*n* = 36/257). No significant correlation was observed between restless behavior and cognitive scores (e.g., TIQ, VIQ) or emotional–behavioral scales.

### 3.2. Cognitive Profiles

#### 3.2.1. Preschool Cognitive Performance

The preschool sample was administered the WPPSI-III in the 4–7.3 years age version for 71.6% (*n* = 53/74) of the children and the 2.6–3.11 years age version for 28.3% (*n* = 21/74) of the children.

The group of children administered WPPSI-III in the 2.6–3.11 years age version had a mean age of 2.84 years (SD 0.36), while the group administered WPPSI-III in the 4–7.3 years age version had a mean age of 4.53 years (SD 0.72).

The average TIQ of the group of children aged 2.6–3.11 years was 120 (SD 5.92). They showed the highest mean weighted scores on the ‘Object Reconstruction’ subtest (13.9, SD 2.34) and the lowest on the ‘Drawing with Cubes’ subtest (12.1, SD 2.32). Furthermore, they had the highest mean scores on the VIQ (118, SD 8.05) and the lowest on the PIQ (117, SD 7.05).

The average TIQ of the group of children aged 4–7.3 years was 123 (SD 6.99). They obtained the highest mean weighted scores on the ‘Vocabulary’ subtest (14.1, SD 2.09) and the lowest on the ‘Image Naming’ subtest (11.4, SD 1.99). Furthermore, they had the highest mean scores on VIQ (118, SD 8.61) and the lowest on the PSQ (113, SD 14.2). In this preschool group, males significantly outperformed females in the Receptive Vocabulary subtest (median score = 13, IQR = 3 vs. 12, IQR = 3; *p* = 0.023, d = 0.35).

#### 3.2.2. School-Age Cognitive Performance

**Figure 1:**
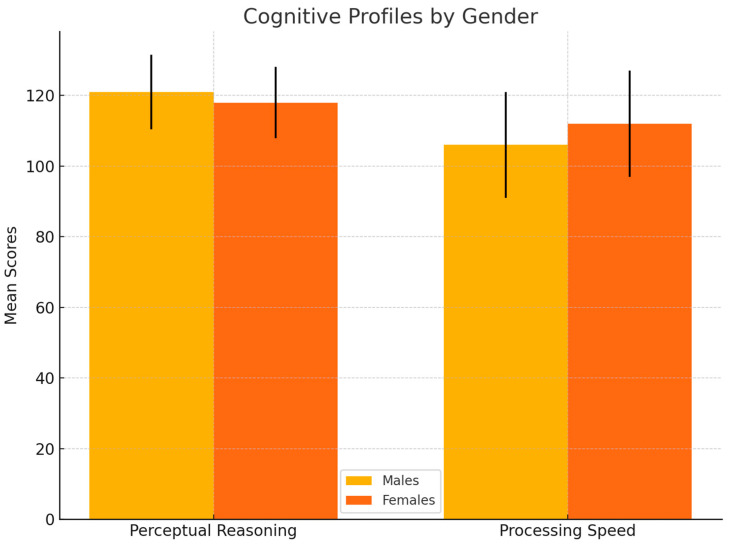
Cognitive profiles by gender.

The average TIQ of the school-aged sample, who completed the WISC-IV, was 120 (SD 5.76). They obtained the highest mean weighted scores on the ‘Similarities’ subtest (13.5, SD 2.54) and the lowest on the ‘Digit Span’ and ‘Letter-Number Sequencing’ subtests (11, SD 2.47 and 11, SD 2.59, respectively). Furthermore, they showed the highest mean scores on the VC index (121, SD 11.5) and the lowest on the WM index (106, SD 13.6). Among school-aged participants, males exhibited significantly higher scores in PR (mean PR = 121, SD = 10.6 vs. 118, SD = 10.1; *p* = 0.032, q = 0.0448), while females scored higher in PS (median score = 112, IQR = 15 vs. 106, IQR = 15; *p* = 0.021, q = 0.0367). These differences remained statistically significant after controlling for multiple testing using the Benjamini–Hochberg procedure. Figure 1 illustrates these cognitive profiles by gender, highlighting significant differences in perceptual reasoning and processing speed.

The descriptive statistics of the quotients of the cognitive level instruments (Wechsler scales) are detailed in Table 2.

Mean scores (± SD) on perceptual reasoning and processing speed indices are shown for males and females. Statistically significant differences emerged for both indices (q < 0.05 after Benjamini–Hochberg correction).

### 3.3. School Learning Skills Assessment

#### 3.3.1. Descriptive Performance in Learning Tasks

Regarding reading skills, 52% (*n* = 87/167) of the children performed within the normal range for speed, 45% (*n* = 77/170) for correctness, and 59% (*n* = 101/169) for comprehension.

In the dictation test for writing ability, 47.3% (*n* = 81/171) of subjects scored below the norm, with 18% (*n* = 32/171) requiring attention and 28% (*n* = 49/171) needing immediate intervention.

In arithmetic operations, 41.8% (*n* = 59/141) of participants scored below the norm, with 24% (*n* = 34/141) requiring attention and 17% (*n* = 25/141) needing immediate intervention.

**Figure 2:**
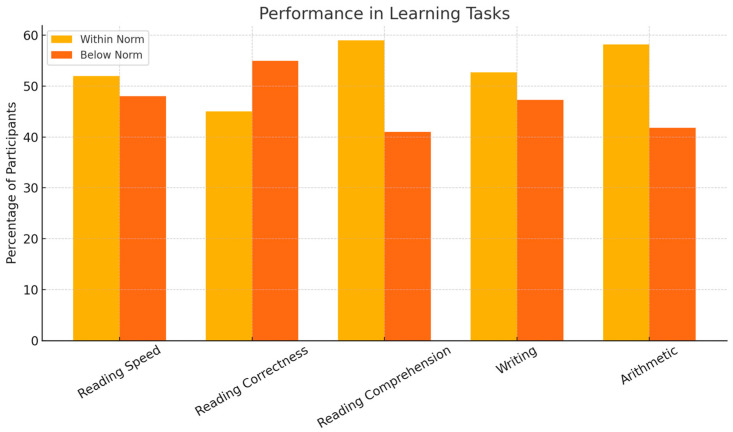
Performance in learning tasks.

Figure 2 presents the distribution of performance in learning tasks. Notably, a substantial subgroup exhibited difficulties in writing, arithmetic, reading correctness, and comprehension, consistent with the observed cognitive discrepancies.

#### 3.3.2. Associations Between Learning Outcomes and Cognitive Profiles

Subjects scoring within the normal range for reading speed achieved significantly higher median scores on the Vocabulary subtest (13, IQR 3 vs. 11.8, IQR 4; *p* = 0.029, q = 0.0452, d = 0.38). Similarly, those with normal performance for reading correctness scored higher on the Vocabulary subtest (13, IQR 3 vs. 11, IQR 4; *p* = 0.038, q = 0.0457, d = 0.56). Interestingly, lower performance in reading correctness correlated with higher scores on the PS index (106, IQR 21 vs. 112, IQR 13; *p* = 0.045, q = 0.0460, d = −0.34).

In reading comprehension, children scoring within the normal range had significantly higher scores on the Digit Span subtest (11, IQR 2 vs. 9.5, IQR 2.75; *p* = 0.007, q = 0.0245, d = 0.63) and the VC index (122, SD 10.8 vs. 114, SD 12.5; *p* = 0.001, q = 0.0070, d = 0.69). In arithmetic, those within the normal range achieved significantly higher scores on the TIQ index (119, IQR 7.25 vs. 117, IQR 4; *p* = 0.020, q = 0.0367, d = 0.33).

A Benjamini–Hochberg correction for false discovery rate was applied to all reported group comparisons and correlation analyses. After adjustment, all significant findings remained robust, including gender differences in cognitive profiles (PR: *p* = 0.032, q = 0.0448; PS: *p* = 0.021, q = 0.0367) and the links between cognitive abilities and academic performance (reading correctness and PS: *p* = 0.045, q = 0.0460; reading comprehension and Digit Span: *p* = 0.007, q = 0.0245; reading comprehension and VC: *p* = 0.001, q = 0.0070; arithmetic performance and TIQ: *p* = 0.020, q = 0.0367). All q-values remained below the 0.05 threshold, reinforcing the robustness of these findings.

The complete distribution of subjects in the reading, writing and calculation criteria bands is shown in Table 3.

Percentage of participants performing within normative ranges versus below the norm in reading speed, reading correctness, reading comprehension, writing, and arithmetic tasks. A substantial proportion exhibited difficulties in writing and arithmetic.

### 3.4. Emotional and Behavioral Problems

#### 3.4.1. Descriptive Data on Emotional–Behavioral Functioning

On the CBCL scale, no clinically significant T-scores emerged in the sample. The preschool group had the highest mean T-scores on the Internalizing scale in both age groups (56.2, SD 11.4 for 2.6–3.11 years; 52.2, SD 10.6 for 4–7.3 years).

Among school-aged children, the highest mean T-scores were also observed on the Internalizing scale (56.7, SD 11.4). Full descriptive statistics of the T-scores of the CBCL Total Problems, Internalizing, Externalizing scales are given in Table 4.

#### 3.4.2. Association Between Emotional–Behavioral Difficulties and Cognitive Performance

**Figure 3:**
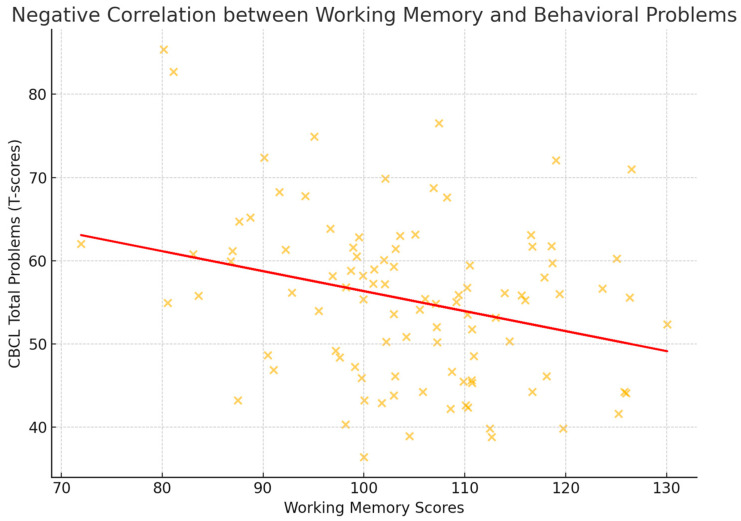
Negative correlation between working memory and behavioral problems.

A weak but statistically significant negative correlation emerged between CBCL Total Problems (6–18 years version) and working memory scores (r = −0.13, *p* = 0.046, q = 0.0460), suggesting that greater emotional–behavioral difficulties may be associated with lower working memory performance. Figure 3 visualizes this association; however, given the modest effect size, the result should be interpreted with caution.

Scatterplot depicting the association between working memory scores (WISC-IV) and CBCL Total Problem scores. The regression line illustrates the modest inverse relationship (r = −0.13, q = 0.0460).

## 4. Discussion

This study represents, to our knowledge, one of the first large-scale investigations exploring cognitive, academic, and emotional–behavioral profiles of above-average cognitive functioning children and adolescents within an Italian clinical setting.

By adopting an inclusive operational approach (IQ > 114), this research captures a broader and more clinically representative population, contributing to the limited body of evidence on the interplay between cognitive abilities, learning outcomes, and emotional characteristics in non-Anglophone contexts. This dimensional perspective reflects real-world challenges faced by high-functioning children, including those at risk of twice-exceptionality, and complements existing studies traditionally focused on strictly defined gifted cohorts.

By focusing on a Mediterranean population, often underrepresented in giftedness research, our work contributes to expanding the literature beyond strictly selective, Anglo-Saxon samples. Furthermore, by integrating cognitive assessments, academic outcomes, and emotional–behavioral characteristics within the same cohort, we provide a multidimensional perspective on giftedness that reflects both strengths and vulnerabilities. This approach is particularly relevant for the identification of twice-exceptional profiles and for informing tailored interventions within clinical and educational contexts.

The findings of this study provide valuable insights into the cognitive, emotional, and learning profiles of intellectually gifted high-functioning Italian children and adolescents, particularly highlighting their strengths in verbal abilities and challenges in processing speed and working memory. These results not only support existing research but also offer new perspectives that can inform future practices, policies, and studies.

Our results highlighted that both the preschool and school groups exhibited their highest performance in verbal abilities, with mean verbal comprehension scores exceeding normative expectations. This aligns with prior research indicating that gifted children often display precocious verbal skills [35,36].

The observed lower mean scores in working memory and processing speed, while still within the normative range, reflect intra-individual cognitive discrepancies rather than absolute weaknesses. This pattern is consistent with the literature suggesting that some high-functioning children prioritize accuracy over speed, engaging in heightened self-monitoring that can reduce processing efficiency [16,17]. While some studies suggest that high-functioning children may prioritize accuracy over speed [16,17], this specific mechanism was not directly assessed in our study and should be considered a theoretical hypothesis rather than an empirical conclusion.

Gender differences were evident, with males outperforming females in perceptual reasoning and females excelling in processing speed, suggesting that tailored interventions may be necessary to address these unique patterns. These findings may reflect underlying neurobiological and social factors influencing cognitive development [37,38].

These observed gender differences remained statistically significant after correction for multiple testing (Benjamini–Hochberg procedure), reinforcing the robustness of these effects and underscoring the necessity for gender-sensitive approaches in both educational and psychological support, ensuring that interventions are tailored to individual needs and strengths.

The study also revealed significant challenges in learning-related skills, particularly in orthographic writing and mathematical abilities, despite participants presenting above-average cognitive functioning. These difficulties may be partially explained by relative intra-individual discrepancies in cognitive processes, such as working memory and processing speed, rather than clinically significant deficits.

This pattern aligns with profiles often discussed in the literature on twice-exceptionality [12,13], although our findings should be interpreted as reflecting potential developmental vulnerabilities rather than definitive evidence of co-occurring learning disabilities.

Although these difficulties co-occurred with relative discrepancies in working memory and processing speed, our data do not permit causal inferences regarding the underlying cognitive mechanisms, nor can we exclude the contribution of unmeasured environmental or educational factors, which future studies should explore.

Based on the post hoc power analysis, the study had sufficient power to detect moderate effects, but limited capacity to reliably detect small or borderline associations. Consequently, weak correlations or marginal group differences should be considered preliminary and require replication in larger, more adequately powered studies.

It is important to note that the associations observed between cognitive abilities and academic performance were modest and, in some cases, revealed complex patterns. For example, lower Digit Span scores were linked to reduced reading comprehension, while lower processing speed was paradoxically associated with better reading correctness, suggesting possible strategic trade-offs or compensatory mechanisms.

These findings underscore the heterogeneity of cognitive–academic profiles in high-functioning children and highlight the need for nuanced, individualized interpretations. Addressing these challenges through tailored interventions is critical for ensuring that these students reach their full potential.

The application of the Benjamini–Hochberg correction confirmed the robustness of the main findings, with all reported associations maintaining statistical significance after adjustment. This enhances the credibility of the observed relationships, particularly regarding gender differences in cognitive profiles and the links between cognitive abilities and academic outcomes.

Regarding emotional and behavioral profiles, all CBCL scores in our sample remained within the non-clinical range, although slightly higher scores were observed on the Internalizing scale of the CBCL. These subclinical tendencies, particularly related to anxiety and perfectionistic traits, are consistent with previous studies describing heightened emotional sensitivity and socio-emotional vulnerabilities in children with above-average cognitive functioning [11,39].

The weak negative correlation between CBCL Total Problem scores and working memory suggests a modest association, but its small magnitude and the absence of clinically significant WM impairments in our sample limit strong conclusions. Future research should employ more sensitive, multi-informant assessments to better explore the emotional characteristics of children with above-average cognitive functioning.

Moreover, it remains unclear whether emotional–behavioral factors influence cognitive functioning or vice versa, highlighting the need for longitudinal studies to clarify these dynamics. Given the normative range of WM scores in our sample, this finding should be interpreted cautiously and does not support definitive claims about cognitive–emotional interplay.

The findings of this study highlight several important implications across educational practices, professional development, policy initiatives, and future research directions. Schools should prioritize the adoption of differentiated instructional strategies that cater to the diverse needs of children with above-average cognitive functioning, particularly those who face learning challenges. This approach ensures that educational programs are inclusive and responsive to the unique profiles of these children.

For example, children showing relative weaknesses in working memory may benefit from targeted interventions focused on metacognitive strategies and structured rehearsal techniques, such as chunking, dual coding, and visual mnemonics, which have proven effective in high-ability learners with executive function vulnerabilities [7,16]. Those with perfectionistic tendencies or internalizing behaviors might respond well to interventions integrating social–emotional learning (SEL) programs or cognitive–behavioral strategies aimed at reducing performance anxiety and maladaptive self-expectations [8,11]. Additionally, students with advanced reasoning skills but orthographic difficulties could benefit from compensatory tools—such as speech-to-text software, structured writing templates, and explicit spelling instruction—especially when these supports are tailored to cognitively gifted profiles [13,24,40].

It is also essential to promote early identification of twice-exceptional profiles, particularly in clinical settings, where subtle cognitive or emotional vulnerabilities may otherwise be overlooked.

In parallel, teachers and psychologists must receive specialized training to effectively identify and support twice-exceptional children, equipping professionals with the tools and knowledge necessary to address the complexities of this population.

From a policy perspective, it is crucial for policymakers to allocate resources towards screening and intervention programs tailored to the specific needs of children with above-average cognitive functioning, with particular attention to underrepresented populations. Such initiatives can bridge existing gaps and create opportunities for a more equitable support system. Lastly, there is a pressing need for longitudinal studies to explore the developmental trajectories of high-functioning children. By examining the interplay between cognitive strengths, emotional well-being, and learning outcomes over time, future research can provide deeper insights into how to optimize the potential of this unique group.

While this study provides valuable insights, several limitations should be acknowledged to contextualize the findings and guide future research. First, the study was conducted in a single clinical setting, which may restrict the generalizability of the results to broader populations. The cross-sectional nature of the study further limits the ability to infer causal relationships, making it important for future studies to adopt longitudinal designs to better capture developmental trajectories.

Additionally, the absence of systematically collected socio-demographic data, such as parental education, socio-economic status, or home environment characteristics, represents an important limitation. These unmeasured factors may have influenced the cognitive, academic, and emotional outcomes observed in our sample, potentially contributing to differences between groups, including the gender-based patterns or learning difficulties reported. In particular, the presence of poor academic achievement in some children with above-average cognitive functioning raises questions about the possible role of socio-economic or environmental disadvantages, which we were unable to evaluate in this study. Future research should integrate comprehensive assessments of family background to better disentangle the contributions of cognitive potential and contextual influences. While the majority of participants came from urban Italian families, this cannot fully rule out the role of socio-family variability. Future studies should incorporate detailed socio-demographic assessments to better disentangle the complex interplay between cognitive profiles, environmental influences, and cultural context.

Additionally, the analytical approach adopted in this study was based on descriptive statistics, group comparisons, and correlational analyses. While suitable for exploratory purposes, this strategy does not allow for the identification of latent cognitive dimensions or the modeling of complex developmental profiles. Future studies should incorporate advanced statistical methods, such as latent structural modeling, factor analysis, or person-centered approaches, to investigate hidden profiles within children with above-average cognitive functioning and to clarify how cognitive, academic, and emotional domains interact.

Furthermore, although age-related subgroup comparisons were performed, we did not apply specific methods to differentiate age effects from potential cohort effects. This limits the developmental interpretation of our findings. Future research employing longitudinal or cohort-sequential designs would be valuable to disentangle these contributions and better characterize developmental trajectories in high-functioning children.

Additionally, the reliance on parent-reported measures for emotional and behavioral assessments may introduce bias. To address this, future research should incorporate self-reports and observational tools to provide a more comprehensive and balanced perspective. Moreover, the absence of a comparison group of non-gifted or typically developing children limits the study’s ability to contextualize the unique cognitive, academic, and behavioral profiles of children with above-average cognitive functioning. Including such a group in future research would provide clearer insights into the distinct characteristics of this population.

Another limitation relates to the determination of sample size, which was based on clinical availability rather than a priori power analysis. This approach may have affected the statistical power of some analyses, highlighting the need for formal power analyses in future research to enhance the reliability and generalizability of results.

Finally, the observed correlation between working memory and emotional–behavioral scores underscores the importance of considering cognitive and emotional factors together when designing interventions for high-functioning children. However, given the modest magnitude of this association and the exploratory nature of our findings, such interpretations should be made cautiously and require replication with more sensitive, multidimensional assessment tools. This finding highlights the need for holistic approaches that integrate cognitive and emotional support to optimize outcomes for this population.

Taken together, the findings of this study offer direct answers to the research questions initially posed. First, cognitively children and adolescents with above-average cognitive functioning demonstrated consistent strengths in verbal reasoning and relative weaknesses in working memory and processing speed, with gender-based differences in perceptual reasoning and processing efficiency. Second, academic achievement was closely linked to specific cognitive abilities: higher verbal comprehension and working memory scores were associated with better reading comprehension and math performance, while lower scores in processing speed and working memory appeared to underpin orthographic difficulties. Third, the emotional and behavioral profile of the sample was characterized by subclinical internalizing tendencies—particularly in preschool and school-age subgroups—with a modest inverse correlation between total behavioral problems and working memory. Lastly, these findings translate into concrete educational and clinical implications, emphasizing the need for targeted interventions such as executive function training, social–emotional skill development, and differentiated academic support.

This study underscores the importance of recognizing the diverse profiles of children and adolescents with above-average cognitive functioning. By addressing their strengths and challenges, we can develop more inclusive and effective approaches to education and support. These findings contribute to a growing body of knowledge that aims to optimize the developmental trajectories of high-functioning individuals.

It is also important to interpret the associations reported between cognitive abilities, academic performance, and emotional outcomes with caution. After considering the potential inflation of Type I error due to multiple testing, the strength of these relationships appears limited. This highlights the multifactorial nature of learning and emotional development, where cognitive potential alone may be insufficient to predict outcomes, particularly when unmeasured socio-economic factors are involved.

## 5. Future Directions: Neuroimaging and the Neuroscience of Giftedness

Building on the preliminary insights of this study, future research should adopt longitudinal designs and comprehensive, multidimensional assessments to better understand the interplay between cognitive abilities, academic achievement, and emotional development in children with above-average cognitive functioning.

Given the exploratory nature of our findings and the modest effect sizes observed, these future investigations should prioritize the inclusion of socio-economic and environmental variables, which were not assessed in the present study but may significantly influence developmental outcomes.

While mechanisms such as self-monitoring or cognitive trade-offs between speed and accuracy have been proposed in the literature to explain performance patterns in high-functioning children [16,17], our study did not directly assess these processes. Future research could empirically test these hypotheses through experimental tasks or process-oriented assessments.

Finally, refining assessment protocols to better identify heterogeneous profiles within this population may contribute to more individualized educational and clinical support, particularly for children exhibiting developmental discrepancies. However, given the current findings, recommendations for addressing twice-exceptionality should be made cautiously and supported by further empirical evidence.

Recent advancements in neuroimaging methodologies, such as functional magnetic resonance imaging (fMRI), diffusion tensor imaging (DTI), and resting-state connectivity analysis, have created new opportunities for investigating the neural correlates of high cognitive potential. Preliminary research indicates that gifted individuals may demonstrate enhanced connectivity within fronto-parietal networks, increased efficiency in neural processing, and distinct patterns of cortical maturation, particularly in regions associated with executive functioning, abstract reasoning, and emotional regulation, such as the prefrontal cortex, anterior cingulate, and temporo-parietal junction [41,42,43,44].

However, most available studies have focused on highly selective samples or used strict IQ-based definitions (e.g., IQ ≥ 130), limiting generalizability to more diverse, clinically referred populations with above-average cognitive functioning, especially within Mediterranean or Southern European contexts.

Although the majority of neuroimaging studies have concentrated on adults or selectively chosen samples of high-functioning youth [45], future investigations should focus on integrating brain-based assessments within developmental, cognitive, and behavioral frameworks, especially in younger populations. This integrative approach could facilitate a more nuanced identification of neurocognitive subtypes of above-average cognitive functioning and clarify the neural foundations of twice-exceptionality, including the influence of learning disabilities or emotional vulnerabilities on the brain functions of gifted individuals.

Moreover, longitudinal neuroimaging studies have the potential to illuminate the dynamic evolution of brain networks associated with advanced cognitive abilities, thereby providing critical insights into the developmental trajectories of high-potential children. The findings from such research may ultimately inform the development of tailored cognitive training programs or neuromodulation interventions designed to enhance strengths while addressing areas of vulnerability.

Adopting a neuroscience-informed perspective on above-average cognitive functioning promises to refine diagnostic criteria and intervention strategies, moving beyond traditional IQ assessments toward a multidimensional and biologically grounded framework for understanding talent and development.

## 6. Conclusions

In conclusion, this study provides a comprehensive overview of the cognitive, emotional, and learning profiles of a sample of Italian children and adolescents with above-average cognitive functioning. Key findings include the following:

- Superior verbal abilities in both preschool and school-aged groups, coupled with relative weaknesses in working memory and processing speed.
- Gender-specific differences in cognitive abilities, with males excelling in perceptual reasoning and females in processing speed.
- Challenges in writing and arithmetic skills, which may reflect underlying cognitive discrepancies rather than definitive learning disorders, warranting careful individualized assessment.
- Elevated internalizing behaviors, such as anxiety and perfectionism, although no clinically significant emotional–behavioral issues were identified.

These findings have practical implications for educators, clinicians, and policymakers. By implementing targeted interventions that address both the strengths and relative cognitive discrepancies of children with above-average cognitive functioning, particularly in areas such as working memory and emotional well-being, we can foster their academic, social, and emotional development more effectively.

However, our findings should not be interpreted as providing direct evidence of twice-exceptionality. Rather, they highlight the presence of heterogeneous developmental profiles that may increase vulnerability to learning or socio-emotional difficulties, particularly in real-world clinical settings. Given the exploratory nature of our analyses, the modest effect sizes observed, and the absence of socio-economic data, these results should be interpreted with caution. Future studies should adopt more comprehensive, multidimensional approaches to capture the interplay between cognitive abilities, socio-environmental context, and developmental outcomes.

Moreover, these results underscore the importance of considering twice-exceptionality and emotional difficulties as possible dimensions of vulnerability, rather than making categorical assumptions, especially in clinical settings where children may present complex, multidimensional profiles.

It is also important to note that no clinically significant emotional–behavioral problems emerged in our sample. The observed tendencies, such as elevated internalizing scores, suggest potential areas of vulnerability but do not establish a systematic link between above-average cognitive functioning and emotional difficulties.

Furthermore, future research should focus on longitudinal studies and diverse populations to deepen our understanding of high cognitive potential and its associated challenges and strengths.

The results of this study contribute to the broader discourse on above-average cognitive functioning, emphasizing the need for a nuanced and multidimensional approach to identifying and supporting gifted children and adolescents.

## Figures and Tables

**Table 1:** Demographic characteristics of the sample.

Variables	Total Sample (*n* = 330) *n* (%) or Mean (SD) *	Preschool Sample (*n* = 74) *n* (%) or Mean (SD) *	School Sample (*n* = 257) *n* (%) or Mean (SD) *
Age, years *	8.68 (3.4)	4.2 (1.42)	9.93 (2.67)
Gender			
*Male*	219 (66.1)	44 (59.4)	175 (68)
*Female*	112 (33.84)	30 (40.5)	82 (32)
Restless Behavior	65 (19.6)	30 (40.5)	36 (14)

* Mean and standard deviation (SD) are reported for continuous variables. Percentages are reported for categorical variables.

**Table 2:** Descriptive statistics of quotients of cognitive level tools (Wechsler scales).

Variables	WPPSI-III (2.6–3.11 yrs) Mean (SD)	WPPSI-III (4–7.3 yrs) Mean (SD)	WISC-IV Mean (SD)
TIQ	120 (5.1)	123 (6.9)	120 (5.7)
VIQ	118 (8.05)	118 (8.61)	-
PIQ	118 (7.3)	116 (10.7)	-
GLI	120 (19.08)	110 (9.9)	-
PSQ	-	113 (14.2)	-
ICV	-	-	121 (11.5)
IRP	-	-	119 (10.3)
WM	-	-	106 (13.6)
PS	-	-	109 (13.5)

TIQ—total intellectual quotient; VIQ—verbal intellectual quotient; PIQ—performance intellectual quotient; GLI—General Language Index; PSQ—processing speed quotient; CV—verbal comprehension; RP—perceptive reasoning; WM—working memory; PS—processing speed.

**Table 3:** Distribution of subjects in the reading, writing and calculation criterion bands.

Variables	*n* (%)
Reading speed (*n* = 167)	
0	32 (19.2)
1	87 (52.1)
2	28 (16.8)
3	20 (12)
Reading accuracy (*n* = 170)	
0	42 (24.7)
1	77 (45.3)
2	36 (21.2)
3	15 (8.8)
Comprehension (*n* = 169)	
0	38 (22.5)
1	77 (45.3)
2	21 (12.4)
3	9 (5.3)
Writing (*n* = 171)	
0	31 (18.1)
1	59 (34.5)
2	21 (12.4)
3	49 (28.7)
Calculation (*n* = 141)	
0	24 (17)
1	58 (41.1)
2	34 (24.1)
3	25 (17.7)

0 = ‘criterion fully achieved’; 1 = ‘sufficient performance’; 2 = ‘demand for attention’; 3 = ‘demand for immediate intervention’.

**Table 4:** Descriptive statistics of the T-scores of the CBCL Total Problems, Internalizing, Externalizing scales.

Variables	2.6–3.11 Years Age Group Mean (SD)	4–7.3 Years Age Group Mean (SD)	School Group Mean (SD)
Total Problems	52.4 (11.5)	50.2 (10.3)	53.3 (11.0)
Internalization	55.4 (11.7)	52.5 (10.6)	56.7 (11.4)
Externalization	48.5 (9.57)	50.1 (10.4)	51.0 (10.5)

## Data Availability

The data presented in this study are available on request from the corresponding author. The data are not publicly available due to privacy and ethical restrictions.
